# Flow-Compensated vs. Monopolar Diffusion Encodings: Differences in Lesion Detectability Regarding Size and Position in Liver Diffusion-Weighted MRI

**DOI:** 10.3390/tomography11100106

**Published:** 2025-09-23

**Authors:** Alessandra Moldenhauer, Frederik B. Laun, Hannes Seuss, Sebastian Bickelhaupt, Bianca Reithmeier, Thomas Benkert, Michael Uder, Marc Saake, Tobit Führes

**Affiliations:** 1 Institute of Radiology, University Hospital Erlangen, Friedrich-Alexander-Universität Erlangen-Nürnberg, Maximiliansplatz 3, 91054 Erlangen, Germany; frederik.laun@uk-erlangen.de (F.B.L.); hannes.seuss@klinikum-forchheim.de (H.S.); sebastian.bickelhaupt@uk-erlangen.de (S.B.); bianca.reithmeier@fau.de (B.R.); michael.uder@uk-erlangen.de (M.U.); marc.saake@uk-erlangen.de (M.S.); tobit.fuehres@fau.de (T.F.); 2 Department of Radiology, Klinikum Forchheim—Fränkische Schweiz, Krankenhausstraße 10, 91301 Forchheim, Germany; 3 MR Application Predevelopment, Siemens Healthineers AG, 91052 Erlangen, Germany; benkert.thomas@siemens-healthineers.com

**Keywords:** diffusion-weighted imaging, flow-compensated, signal dropout, liver lesions, lesion detection

## Abstract

**Background/Objectives**: Diffusion-weighted imaging (DWI) of the liver is prone to cardiac motion-induced signal dropout, which can be reduced using flow-compensated (FloCo) instead of monopolar (MP) diffusion encodings. This study examined differences in lesion detection capabilities between FloCo and MP DWI and whether visibility depends on lesion size and position. **Methods**: Forty patients with at least one known or suspected focal liver lesion (FLL) underwent FloCo and MP DWI. For both sequences, b = 800 s/mm^2^ images were used to manually segment FLLs, which were then sorted by size and location (liver segment). The number of detected lesions, the sensitivity, and the contrast-to-noise ratio (CNR) were calculated and compared across sequences, sizes, and locations. **Results**: Significantly more lesions were detected using FloCo DWI compared to MP DWI (1211 vs. 1154; *p* < 0.001). In total, 1258 unique lesions were detected, 104 of which were identified only by FloCo DWI, and 47 of which only by MP DWI. The sensitivities of FloCo DWI and MP DWI were 96.3% (95% CI: 95.1–97.2%) and 91.7% (95% CI: 90.1–93.2%), respectively. The largest additional lesion found with only one of the two sequences measured 10.9 mm in FloCo DWI and 8.2 mm in MP DWI. In relative numbers, more additional FloCo lesions were found in the left liver lobe than in the right liver lobe (6.4% vs. 3.5%). The lesion CNR was significantly higher for FloCo DWI than for MP DWI (*p* < 0.05) for all evaluated size intervals and liver segments. **Conclusions**: FloCo DWI appears to enhance the detectability of FLLs compared to MP DWI, particularly for small liver lesions and lesions in the left liver lobe.

## 1. Introduction

The treatment for malignant tumors depends on the extent of the disease. Since tumors often metastasize to the liver, accurate detection of liver involvement is crucial [1,2,3,4]. Diffusion-weighted MRI (DWI) has proven effective at detecting focal liver lesions (FLLs) and is, therefore, commonly used for staging [5,6,7].

While lower DWI b-values are suitable for detecting FLLs, higher b-values help characterize them as benign or malignant [8,9]. However, with the higher diffusion weighting used for liver DWI in a clinical setting (e.g., b = 800 s/mm^2^), the pulsatile motion of the heart negatively affects the image quality by generating motion artifacts, particularly in the left liver lobe due to its proximity to the heart [9,10,11,12]. This artificial signal loss may make FLLs difficult to detect or even undetectable [13]. Different technical approaches have been suggested to address this issue, including electrocardiogram (ECG) triggering [14,15,16,17,18] and weighted signal averaging [11,19].

Besides cardiac gating and post-processing techniques, modifying DWI sequences with velocity-compensated (commonly named “flow-compensated (FloCo)”) diffusion encodings has been tested to improve the described issue. FloCo DWI can reduce signal dropouts in the liver [20,21] and, thus, improve FLL detection. Laun et al. dichotomized lesions into two categories, small and large. Son et al. used a five-point Likert scale to analyze lesion conspicuity. By comparing their detection rate with FloCo and monopolar (MP) DWI, both studies resulted in overall improved image quality and lesion depiction with FloCo DWI [22,23].

However, no studies have reported on how size and position influence detectability of FLLs between conventional MP and FloCo diffusion encodings in oncological patients. Therefore, this study compared FloCo and MP DWI in regard to FLL visibility and the number of missed lesions, sorting them by size and position to better understand the potential impact of FloCo DWI in clinical settings.

## 2. Materials and Methods

The study was approved by the local Institutional Ethics Committee (Study Number: 276_19 B, Date of Approval: 7 August 2019) and conducted according to the Declaration of Helsinki. Written informed consent was obtained from all patients. The privacy rights of the patients have been observed at all times. The data used in this study has previously been employed (see [22,24]). In the previous evaluation by Laun et al., only two size categories were considered (diameter <1 or >1 cm), and the lesions were neither counted nor segmented [22].

### 2.1. Study Population

This retrospective study examined 40 patients aged ≥18 years, consecutively recruited between January and August 2020, during the clinical workflow, as part of a prospective study [22]. Inclusion criteria were the presence of at least one known or suspected liver lesion (stage IV cancer) and an expected tolerance to a prolonged MRI examination incorporating a DWI sequence. The exclusion criteria were the presence of active or ferromagnetic implants, claustrophobia, and the use of sedative medications.

### 2.2. MRI

All scans were performed using a research application sequence on a clinical 1.5 T MRI scanner (MAGNETOM Aera; Siemens Healthineers, Erlangen, Germany), with a vendor-provided 18-channel anterior body matrix coil in combination with a vendor-provided 32-channel spine array coil. Two diffusion-weighted sequences were acquired (“MP” and “FloCo”), which differed only in the order of gradient moment nulling. The technical details of the acquisitions performed are listed in Table 1 (adapted from [22]). Moreover, a standard clinical liver MRI protocol was conducted for each patient, which included a T2-weighted HASTE sequence and fat-saturated T1-weighted GRE sequences, one before and several after the administration of the contrast agent. The contrast agent was given after the diffusion sequences. Please note that, throughout this paper, the term “flow-compensated” is used according to the scanner’s label of the respective sequence, yet essentially a velocity-compensated sequence was applied.

### 2.3. Image Analysis

#### 2.3.1. Segmentation

Segmentations were performed using the software MITK (Medical Interaction Toolkit, v.2022.04) by a final-grade medical student (A.M.), guided and supervised by a board-certified radiologist with over 15 years of experience in abdominal imaging (M.S.). Blinding of the acquisition type was not implemented, since the two sequences could be easily distinguished from the image impression.

Images were acquired with b = 50 s/mm^2^ and 800 s/mm^2^, but only b800-images were used for evaluation, as the aim of this study is to reduce the signal dropout that appears in diffusion-weighted images with a higher b-value. For each patient, the MP images were inspected first. All identified FLLs were manually segmented (3D segmentations) and numbered. Next, the FloCo images were inspected, and again, detected FLLs were manually segmented. Corresponding FLLs were assigned the same numbers, and additional lesions identified were labeled as “only_floco.” MP lesions with no corresponding FloCo lesions were relabeled as “only_mp.” In case of doubt, due to FLLs with poor visibility in one sequence but good visibility in the other, FLLs were segmented in both sequences and deemed detectable instead of being counted as additional lesions in only one sequence. In order to ensure a qualitative comparison, both sequences of the same patient were set to identical windowing.

FLLs were always completely segmented, potentially including central necrosis. To avoid partial volume effects, and therefore alteration of the FLLs’ mean signal intensities, the edges of FLLs were excluded from the segmentation. If neighboring lesions had grown to such an extent that the borders had become undetectable, they were counted as one FLL. Lesions whose appearance indicated benignity were excluded.

If an FLL was detected, its correlating anatomic liver segment (I–VIII) was noted. When an FLL crossed the borders of liver segments, the liver segment with the largest proportion of the FLL was used in the evaluation.

Additionally, for each patient, in both sequences, respectively, a single 10 cm^2^ reference region of healthy liver parenchyma was manually segmented on the central slice of each stack containing 39 slices (see Table 1), excluding vessels and lesions, except for one case in which the reference region was not segmented on the central slice since there was hardly any liver parenchyma left to see; it was instead drawn on a different slice. All reference regions were segmented in either segment VIII or V, depending on anatomy. These segmentations were later used to calculate the contrast-to-noise ratio (CNR; see below).

#### 2.3.2. Evaluation

FLLs that appeared in both sequences were counted as one lesion. FLLs that appeared in only one of the two sequences counted as additional lesions.

The FLLs of all patients were sorted by size into seven categories, based on the number of voxels: 1–10, 11–25, 26–50, 51–100, 101–250, 251–500, and ≥501. Assuming the FLLs to be geometric spheres, the corresponding diameter intervals (in millimeters) were calculated based on the equations $V_{lesion}=\frac{4}{3}\times \pi \times r_{lesion}^{3}$ and $V_{lesion}={x\times V}_{Voxel}$ with x representing the number of voxels and $V_{voxel}=1.56^{2}\times 6\;{mm}^{3}$. Note that 6 mm represents slice thickness (5 mm) plus slice distance (20% = 1 mm). This results in the following equation.

$$d_{lesion}=2\times r_{lesion}=2\times \sqrt[{3}]{\frac{3\times x\times 1.56^{2}\times 6}{4\times \pi }}mm$$

The calculation resulted in the following size limits for the seven categories (in millimeters): ≤6.5, 6.6–8.8, 8.9–11.1, 11.2–14.0, 14.1–19.1, 19.2–24.0, and ≥24.1.

For FLLs that were detected with both sequences, the lesion size measured with the FloCo DWI was deemed to be closer to reality due to its improved ability to depict FLLs at higher b-values [22], and thus was used as the measured size for both sequences (FloCo and MP). This approach was only used to prevent the same FLL from being placed into different size categories due to seemingly different sizes based on its appearance in the respective sequences, which would confound the statistical analysis.

In order to quantify the number of additional lesions, their difference (N_Δ_) was calculated by subtracting the additional lesions found with the MP DWI (N_only_mp_) from the additional lesions found with the FloCo DWI (N_only_fl_): N_Δ_ = N_only_fl_ − N_only_mp_. N_Δ_ > 0 indicates that more additional lesions were detected with the FloCo DWI, whereas N_Δ_ < 0 means that more additional lesions were found with the MP DWI.

Further calculations included the number of lesions found in both sequences (N_both_), as well as all lesions counted (N_total_all_ = N_only_mp_ + N_only_fl_ + N_both_). For relative comparison, the ratios of additional lesions (N_only_mp_, N_only_fl_, and N_Δ_) to all FLLs (N_total_all_) were calculated.

The CNR was calculated using the respective reference segmentation of the liver parenchyma (see above). For each FLL, the CNR was calculated as follows [25]:

$$CNR=\frac{S_{0}-S_{ref}}{\sigma_{ref}},$$

where S_0_ is the mean signal of the lesion, S_ref_ is the mean signal of the reference region, and σ_ref_ is the standard deviation of the reference region. Negative CNR values for barely visible FLLs were included in the analysis. For every additional lesion found in one of the two sequences, the respective lesion CNR was set to 0 for the other sequence.

The results of the FLLs’ numbers, their respective ratios to N_total_all_, if applicable, as well as their CNR were sorted by size category (see above) and liver segment.

### 2.4. Statistical Analysis

Statistical analyses were performed using MATLAB (release 2022b; MathWorks, Inc., Natick, MA, USA). The null hypothesis *H*_0_ states that there is no difference in the detection of FLLs between the two DWI sequences. In order to investigate whether *H*_0_ is true, several tests to evaluate the differences in lesion quantity and CNR were executed.

To compare the number of found FLLs between the two sequences, particularly the quantity of additional lesions, McNemar’s test for paired nominal data was used. The two possible outcomes were defined as “visible” and “not visible”. A *p*-value < 0.05 was considered significant. Additionally, the sensitivity and confidence intervals (CIs) using the Clopper–Pearson method with a 95% confidence level for lesion detection was calculated under the assumption that each FLL was detected by at least one sequence and no FLLs were missed, with N_both_ as “true positives” as well as N_only_mp_ and N_only_fl_ as “false negatives”, respectively. Other statistical parameters often mentioned along with sensitivity, such as specificity, and positive or negative predictive value, could not be calculated since, without confirmation, e.g., via biopsy, whether a visible FLL actually is a malignant lesion, the evaluation of “false positives” and “true negatives” was not feasible.

The Shapiro–Wilk test was used to determine whether the CNR values were normally distributed. Since they were not, the Wilcoxon signed-rank test for paired samples was used to compare CNRs between the FloCo and MP DWI. Again, the significance level was set to 0.05.

## 3. Results

### 3.1. Study Population

Forty patients were examined in this study (27 males, 13 females; age range: 34–74 years, mean age: 60 ± 9 years). The patients’ primary diseases varied, encompassing neuroendocrine tumors (52.5%), colorectal cancer (25.0%), thyroid cancer (10.0%), melanoma (5.0%), pancreatic cancer (2.5%), non-small cell lung cancer (2.5%), and mixed adenoneuroendocrine carcinoma (2.5%).

### 3.2. Representative Images

**Figure 1:**
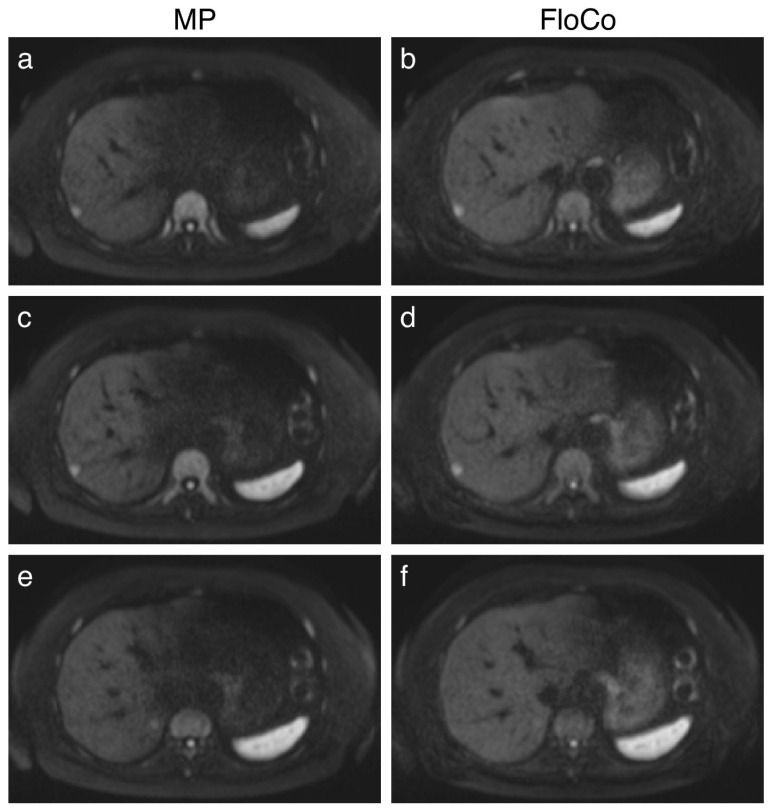
Liver diffusion-weighted MRI (b = 800 s/mm^2^) of a patient with metastatic colorectal carcinoma. Examples of signal dropout. (**a**,**c**,**e**) Monopolar (MP) DWI, three adjacent slices; (**b**,**d**,**f**) flow-compensated (FloCo) DWI, three adjacent slices. Note the reduced ability to determine the liver’s border due to signal dropout in the MP images. Note that a lesion is more clearly visible in the MP image (**e**) than in the corresponding FloCo image (**f**), consistent with our finding that in some cases MP provided greater lesion conspicuity.

**Figure 2:**
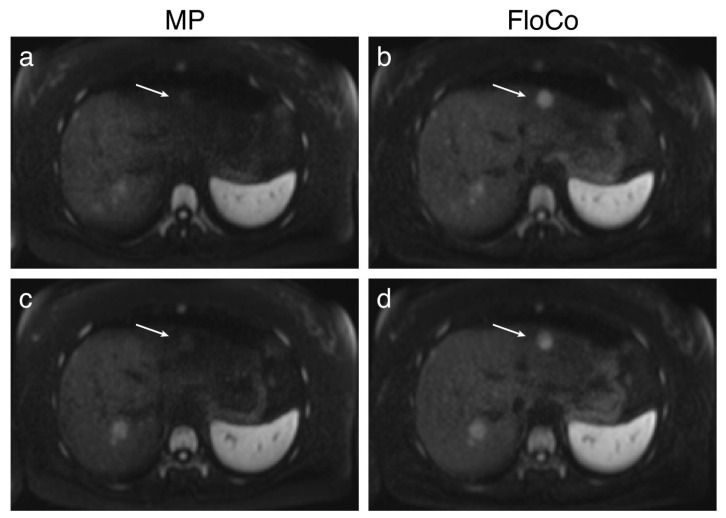
Liver diffusion-weighted MRI (b = 800 s/mm^2^) of a patient with metastatic neuroendocrine tumor. Examples of poorly visible focal liver lesions (FLLs). (**a**,**c**) Monopolar (MP) DWI, two adjacent slices; (**b**,**d**) flow-compensated (FloCo) DWI, two adjacent slices. The arrows highlight an FLL in segment II that is clearly noticeable in the FloCo image, but barely visible in the MP image. However, the FLL was labeled as “visible in both sequences”.

**Figure 3:**
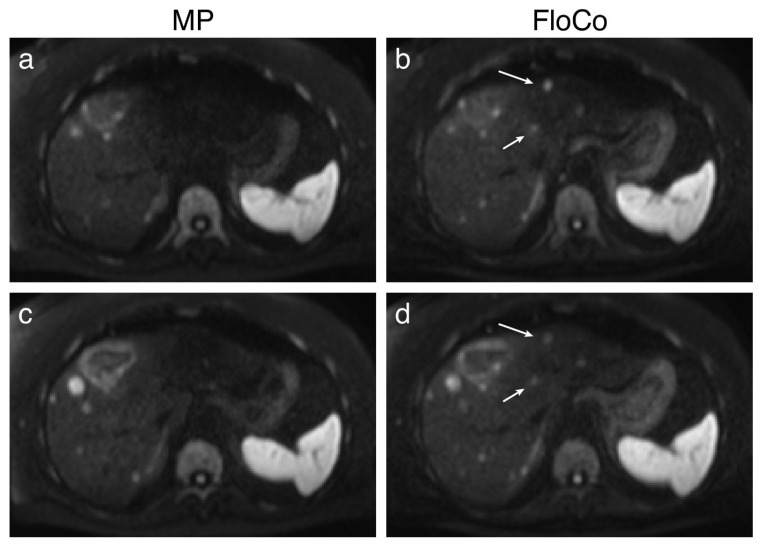
Liver diffusion-weighted MRI (b = 800 s/mm^2^) of a patient with metastatic neuroendocrine tumor. Examples of focal liver lesions (FLLs) visible only in flow-compensated (FloCo) DWI. (**a**,**c**) Monopolar (MP) DWI, two adjacent slices; (**b**,**d**) FloCo DWI, two adjacent slices. The arrows highlight two FLLs in segments II and IV that were not detectable with MP DWI. In this example, the FLLs were labeled as “only_floco”.

Figure 1, Figure 2 and Figure 3 present examples of the difference between the MP and FloCo DWI (b-value = 800 s/mm^2^). Figure 1 presents a representative image of a signal dropout in the MP DWI and its reduction in the FloCo DWI. Figure 2 and Figure 3 present examples of a signal dropout in the left liver lobe causing issues with finding the FLL; the marked FLLs in Figure 2, located in segment II, are visible with both sequences, but are much harder to detect with MP DWI, whereas the two FLLs in Figure 3, located in segments II and IV, are detectable with FloCo DWI, but not with MP DWI.

### 3.3. Quantitative Analysis

Significantly more FLLs were detected by FloCo DWI than by MP DWI (1211 vs. 1154, *p* < 0.001). Of the 1258 unique FLLs identified, 1107 were detectable with both sequences, 104 were detectable only with FloCo DWI, and 47 were detectable only with MP DWI.

The sensitivity for FloCo DWI was 96.3% (95% CI: 95.1–97.2%); the sensitivity of MP DWI was 91.7% (95% CI: 90.1–93.2%).

The number of FLLs detected per patient ranged from 1 to 191 (median: 11) in FloCo DWI and from 1 to 187 (median: 12) in MP DWI. Since 1107 FLLs were segmented twice, once in FloCo images and once in MP images, 2365 segmentations were performed. Given the number of scanned patients, this equates to a mean of 30.3 FLLs, including 2.6 additional lesions, per patient for FloCo DWI and 28.9 FLLs, including 1.2 additional lesions, per patient for MP DWI.

The largest FLL detected was in segment IV, was visible with both sequences, and had a diameter of 104.1 mm (size: 40,491 voxels). The largest additional lesion with FloCo DWI was located in segment II and had a diameter of 10.9 mm (size: 46 voxels), and with MP DWI, was located in segment V and had a diameter of 8.2 mm (size: 20 voxels). For FLLs seen in both modalities, the sizes measured in FloCo were slightly larger, with a mean diameter difference of 0.4 mm. Regarding the number of lesions, the largest relative difference per patient was 400%, with five lesions in FloCo compared to one lesion in MP. The largest absolute difference per patient was 13 lesions, with 167 lesions in FloCo versus 154 lesions in MP.

#### 3.3.1. Size-Based Evaluation

Table 2 shows the number of FLLs, the difference in additional lesions (N_Δ_), their ratio to the number of all FLLs (N_total_all_), and the sensitivities of FloCo and MP DWI for each size category. Additional lesions were only detected at smaller sizes (diameter ≤11.1 mm with FloCo DWI and ≤8.8 mm with MP DWI). The number of additional lesions decreased with size. Therefore, most additional lesions belonged to the smallest size category (diameter ≤6.5 mm). A decrease in the N_Δ_/N_total_all_ ratio and an increase in the sensitivity rate in both sequences was also observed. In the smallest size category, the sensitivity rates of MP DWI and FloCo DWI differ the most. No additional lesion with a diameter >11.1 mm was detected.

**Figure 4:**
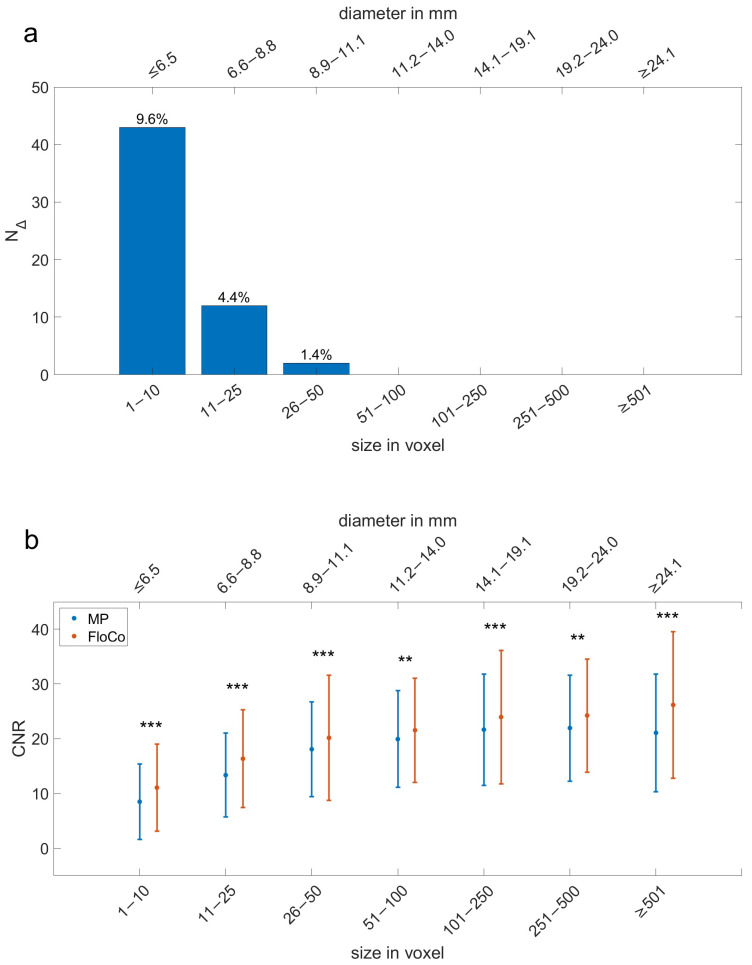
The difference in the number of additional lesions (N_Δ_) and contrast-to-noise ratio (CNR) by size category. Size ranges are shown in terms of lesion diameter (in millimeters; upper *x*-axis) and the number of voxels (lower *x*-axis). (**a**) N_Δ_ is positive and decreases over the first three categories; the percentages describe N_Δ_ in relation to N_total_all_ in each size category. (**b**) The mean and standard deviation of each sequence are shown side by side for each size category. The mean of each size category is presented as a dot, and the standard deviation is shown as error bars. The *p*-value of the CNR differences is presented: ** = *p* < 0.01, *** = *p* < 0.001.

Figure 4a shows the differences in additional lesions (N_Δ_) by size. Once more, the smallest size range presents the largest N_Δ_. In the figure, the N_Δ_/N_total_all_ ratio is shown above each respective bar and decreases with lesion size.

Figure 4b compares the means and standard deviations of the CNRs for MP and FloCo DWI. They are higher for FloCo DWI in all seven size categories. In Table 2, the N_total_all_ column shows the number of FLLs accounted for in each size category. The differences in the CNRs between MP and FloCo DWI were significant for all size ranges. Overall, the CNR increased with size.

#### 3.3.2. Location-Based Evaluation

Regarding the location of the lesions, 438 (FloCo) and 409 (MP) were detected in the left liver lobe (segments I–IV), whereas 773 (FloCo) and 745 (MP) were detected in the right liver lobe (segments V–VIII).

Table 3 presents an overview of the counted FLLs per liver segment. Additional lesions were detected in all eight liver segments. The most additional lesions were counted in segment V (*n* = 16) with MP DWI and segment VIII (*n* = 29) with FloCo DWI. Considering the percentages of additional lesions (N_only_fl_, N_only_mp_) relative to all FLLs within their respective segment (N_total_all_), the largest fraction of additional lesions was in segment VI (7.2%) for MP DWI and segment II (11.7%) for FloCo DWI. The sensitivities of MP DWI and FloCo DWI are also displayed. In FloCo DWI, there were higher sensitivity rates in the liver segments II, IV, VII, and VIII, whereas in MP DWI, the highest sensitivity was in segment I. The largest difference in sensitivity rates was found in segment II, with 100% in FloCo DWI compared to 88.3% in MP DWI.

**Figure 5:**
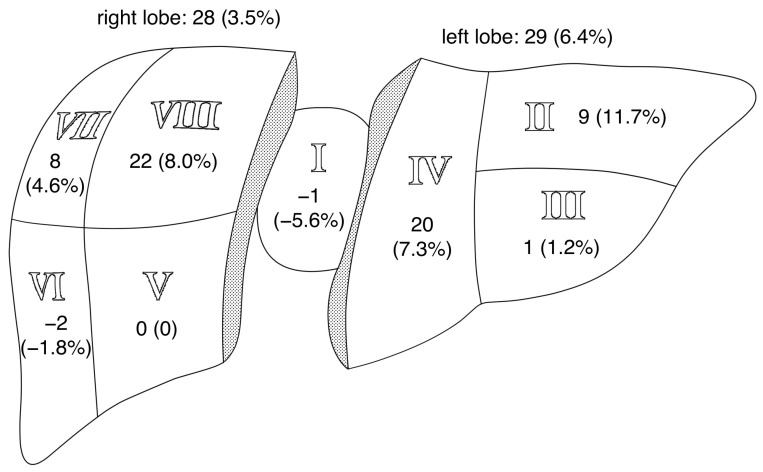
The N_Δ_ for each segment and lobe. The N_Δ_/N_total_all_ ratio is given in brackets, with the respective values for the right lobe (segments V–VIII) and left lobe (segments I–IV) written at the top.

Figure 5 shows the absolute number of N_Δ_ for each segment and lobe, and its ratio to N_total_all_. N_Δ_ was especially large in segment II, the one closest to the heart (>10.0%).

The CNRs for the FloCo and MP DWI are presented for each liver segment in Table 4. They differed significantly for all liver segments (*p* < 0.05). The mean CNRs were higher with FloCo DWI than with MP DWI for each segment.

## 4. Discussion

This study compared the number and CNR of liver lesions measured with conventional MP and FloCo DWI at b = 800 s/mm^2^, stratifying the results by size and location. FloCo DWI was more sensitive than MP DWI in detecting lesions, with significant differences observed for all size categories and liver segments, particularly for smaller lesions close to the heart.

While the lesion CNRs differed significantly between the FloCo and MP DWI, they had large standard deviations, and some CNR values were negative. The reason is that the CNR was calculated using one reference region of 10 cm^2^ per patient for all lesions. This region was always segmented in the right liver lobe since it shows less signal loss than the left lobe [10,20,26]. Therefore, FLLs affected by signal dropout (e.g., in segment II) sometimes had a weaker signal intensity than the reference region. This resulted in a negative CNR, which inevitably led to high standard deviations. Noise estimation in this study was based on the standard deviation in reference tissue ROIs of the averaged images, which provides only an approximation of the true CNR. While subtraction of repeated acquisitions would yield a more robust estimate in parallel imaging, we chose this approach to reflect clinical practice, where radiologists interpret averaged images. In this study’s design, one method to reduce the high standard deviations and perhaps even avoid negative CNRs could be to select a region of interest on each slice on which FLLs are detected. This approach was infeasible in this study since there were at least three patients with a very large number of lesions, making finding a 10 cm^2^ region of interest of healthy liver tissue in each slice with FLLs impossible.

Current developments demonstrate the growing clinical value of liver DWI, a technique which makes the detection of FLLs possible while abstaining from contrast agents, as its routine use for detecting malignant liver lesions, especially small ones, is being considered [4,5,27]. This underlines that accurately depicting FLLs for effective and early diagnosis and treatment, in respect of an improved prognosis, remains a key focus. The application of FloCo acquisition techniques has been assessed in different studies with the prospect of reducing the effects of bulk motion in different organs, such as the brain, the pancreas, or the heart [21,28,29]. Instead of quantifying the improved image quality of FloCo DWI in healthy tissue, this study successfully aimed to increase the detection rate of FLLs with FloCo acquisition techniques, and hence provides a possible approach to reduce the signal dropout caused by cardiac motion.

This study has some limitations. Firstly, while its results show an overall improved depiction of FLLs in FloCo DWI, the additional FLLs detected with MP DWI should not be disregarded. In absolute terms, 47 FLLs were undetectable with FloCo DWI and were, therefore, missed compared to 104 that were undetectable with MP DWI. Additionally, in some cases, lesions were more conspicuous in MP DWI than in FloCo DWI. This may be explained by free-breathing acquisition, where variability in breathing pattern and intensity can cause motion-related blurring that affects the two encoding schemes differently. Nevertheless, across the large number of segmented lesions, FloCo consistently provided superior visibility on average. Furthermore, using the same window settings, a subjective reduction in the signal dropout in FloCo images can be observed, but is not wholly removed. Previous studies have proposed combining FloCo encoding with post-processing techniques as a possible solution to this issue [22,24]. Secondly, a specific strength of conventional, high b-value DWI is that the signal of blood vessels is sufficiently suppressed and appears black in the image. However, with FloCo encoding, blood vessels may occasionally appear bright again and, therefore, could be mistaken as FLL [20]. This issue naturally leads to the concern that a bright-appearing blood vessel may be incorrectly segmented and counted as a liver lesion in this study, although manual segmentation was performed with utmost accuracy and checked several times. Some of the additional findings may represent bright blood vessels rather than true lesions; this cannot be definitively excluded and should be considered when interpreting the results. However, Führes et al. have recently shown no significant difference in the bright blood signal between MP and FloCo DWI b800-images [30], mitigating this concern. Thirdly, the detected FLLs were ultimately not histologically tested for malignity, even though FLLs that showed benign characteristics were excluded during the segmentation stage. Hence, the calculations for the sensitivity of each sequence are based on the assumption of the flawless depiction of FLLs in both sequences combined, using the union of both sequences as ground truth and, thus, representing relative detectability of the FLLs. Fourthly, we did not compare the diffusion-weighted images with more commonly used acquisitions, like imaging after the application of liver-specific contrast agents. In particular, the combined usage of DWI and contrast enhancement seems to improve the detection rate of FLLs [31,32]. However, in contrast-enhanced acquisitions, FLLs might also appear isointense because their depiction depends on vascularity [32,33,34], which might lead to FLLs being missed. Fifthly, several studies have shown that different image acquisition techniques that account for the patient’s breathing might reduce artifacts and improve lesion detection [35,36,37,38,39,40]. However, these potential breathing-related artifacts were not considered in this study’s evaluation, which could be a reason for FLLs appearing in only one sequence, or not at all. Sixthly, lesion segmentation was performed by a single reader under expert supervision, which ensured consistency but precluded assessment of inter-operator variability. Future studies with multiple independent readers are warranted to evaluate inter-rater reliability.

To date, several other technical approaches have been proposed to mitigate the described issue. For example, ECG triggering can be used to acquire images at specific time points, synchronized to the cardiac cycle. Its most severe limitation is the significantly prolonged image acquisition time, preventing its routine use in clinical settings [14,16,18]. Riexinger et al. found that acquisition at inspiration, when the coupling between liver and heart might be minimal, did not improve image quality [41]. Post-processing techniques, such as weighted averaging of the DWI, can balance the signal loss using either “gold standard” target images or deep learning by focusing on designated features [11,19,42]. However, Laun et al. stated that post-processing techniques might be challenging to apply since they brighten the signal of blood vessels, which could be ultimately mistaken as FLLs [22]. Führes et al. largely mitigated this issue by introducing an approach to improve the quality of FloCo DWI and increase the lesions’ CNR by applying post-processing techniques to FloCo-acquired images [24]. Later approaches are region-based shot rejection schemes that, besides post-processing techniques, influence signal dropout in the early stages of signal acquisition without sequence modification or combine motion-robust diffusion-weighted gradient waveforms with multi-shot echo-planar MRI (EPI) to reduce image distortion. However, their clinical implementation still seems challenging due to their possibly unpredictable scan time when using multi-shot EPI. Moreover, Lee et al. described presumably new, standard apparent diffusion coefficients (ADCs) for tissues in the abdomen, which would be difficult to adjust to [21,43].

Future studies on detecting FLLs with FloCo DWI might consider post-processing techniques, like Führes et al. [24], and ADC maps for better certainty regarding the malignity of liver lesions [44,45,46,47]. Another, more clinical approach could include a combination of MP and FloCo sequences in the diagnostic process, using DWI acquisitions with multiple b-values to analyze sensitivity, as well as biopsies of additional lesions detected with only FloCo and their impact on the patients’ oncological treatment. Additionally, the usage of FloCo DWI to detect FLL types other than metastases might be a valuable subject for further research.

## 5. Conclusions

FloCo DWI enabled the detection of more lesions than MP DWI, especially for small lesions and lesions close to the heart. Moreover, FloCo DWI yielded a better lesion CNR and, therefore, better conspicuity. As FloCo DWI also missed some lesions, it could be used as a valuable addition to MP DWI rather than a replacement, especially when small liver lesions are suspected and their identification could influence the therapeutic approach.

## Figures and Tables

**Table 1:** The MRI parameters used for the monopolar (MP) and flow-compensated (FloCo) DWI sequences. DWI = diffusion-weighted MRI, EPI = echo-planar MRI, GRAPPA = GeneRalized Autocalibrating Partial Parallel Acquisition, SPAIR = Spectral Adiabatic Inversion Recovery. For the best possible comparison between the two sequences, the same parameters for both acquisitions were used when feasible. The table was adapted from [22].

Parameter	MP	FloCo
Sequence	DWI EPI	
Repetition time (ms)	12,400	
Echo time (ms)	70	
Echo spacing (ms)	0.49	
Maximum gradient strength (mT/m)	45	
Maximum slew rate (T/m/s)	200	
Voxel size (mm^3^)	3.125 × 3.125 × 5 interpolated to 1.56 × 1.56 × 5	
Field of view (read × phase; mm^2^)	400 × 325	
Phase direction	anterior–posterior	
Phase resolution	100%	
b-values (s/mm^2^)	50, 800	
Averages (b50, b800)	1, 4	
Diffusion mode	3-scan trace	
Diffusion scheme	Monopolar (zeroth-order gradient moment nulling)	velocity-compensated (zeroth- and first-order gradient moment nulling)
Matrix	128 × 104	
Number of slices	39 (axial)	
Slice distance	20%	
Parallel imaging	GRAPPA × 2, 24 reference lines	
Partial Fourier	6/8	
Acquisition time (min:s)	3:43	
Bandwidth (Hz/pixel)	2790	
Surface coil intensity correction	Yes, the “pre-scan normalize” option was used	
Fat saturation	SPAIR and gradient reversal	
Acquisition mode	Free breathing	

**Table 2:** The number of lesions in each size category. Size ranges are displayed in voxels, and diameters are displayed in millimeters. N_only_mp_ = additional focal liver lesion (FLL) only detected with monopolar (MP) DWI, N_only_fl_ = additional FLL only detected with flow-compensated (FloCo) DWI, N_both_ = FLL detected with both sequences, N_total_all_ = N_both_ + N_only_mp_ + N_only_fl_; N_Δ_ = N_only_fl_ − N_only_mp_. CI = confidence interval. The ratios of N_only_mp,_ N_only_fl_, and N_Δ_ to N_total_all_ are also shown. The last two columns display the sensitivities with respective CIs of MP DWI and FloCo DWI for each size category.

Size		N_total_all_	N_both_	N_only_mp_	$\frac{N_{\mathrm{only}\_\mathrm{mp}}}{N_{\mathrm{total}\_\mathrm{all}}}$	N_only_fl_	$\frac{N_{\mathrm{only}\_\mathrm{fl}}}{N_{\mathrm{total}\_\mathrm{all}}}$	N_Δ_	$\frac{N_{\Delta }}{N_{\mathrm{total}\_\mathrm{all}}}$	Sen. (95% CI) MP	Sen. (95% CI) FloCo
In Voxels	⌀ in mm										
1–10	≤6.5	446	317	43	9.6%	86	19.3%	43	9.6%	80.7% (76.7–84.3%)	90.4% (87.2–92.9%)
11–25	6.6–8.8	272	252	4	1.5%	16	5.9%	12	4.4%	94.1% (90.6–96.6%)	98.5% (96.3–99.6%)
26–50	8.9–11.1	146	144	0	0	2	1.4%	2	1.4%	98.6% (95.1–99.8%)	100% (97.5–100%)
51–100	11.2–14.0	105	105	0	0	0	0	0	0	100% (96.6–100%)	100% (96.6–100%)
101–250	14.1–19.1	122	122	0	0	0	0	0	0	100% (97.0–100%)	100% (97.0–100%)
251–500	19.2–24.0	62	62	0	0	0	0	0	0	100% (94.2–100%)	100% (94.2–100%)
≥501	≥24.1	105	105	0	0	0	0	0	0	100% (96.6–100%)	100% (96.6–100%)
Summary		1258	1107	47	3.7%	104	8.3%	57	4.5%	91.7% (90.1–93.2%)	96.3% (95.1–97.2%)

**Table 3:** The number of focal liver lesions (FLLs) per liver segment. N_only_mp_ = additional FLL only detected with monopolar (MP) DWI, N_only_fl_ = additional FLL only detected with flow-compensated (FloCo) DWI, N_both_ = FLL detected with both sequences, N_total_all_ = N_both_ + N_only_mp_ + N_only_fl_; N_Δ_ = N_only_fl_ − N_only_mp_. CI = confidence interval. The ratios N_only_mp_, N_only_fl_, and N_Δ_ to N_total_all_ are also shown. The last two columns display the sensitivities with respective CIs of MP DWI and of FloCo DWI for each liver segment.

Liver Segment	N_total_all_	N_both_	N_only_mp_	$\frac{N_{\mathrm{only}\_\mathrm{mp}}}{N_{\mathrm{total}\_\mathrm{all}}}$	N_only_fl_	$\frac{N_{\mathrm{only}\_\mathrm{fl}}}{N_{\mathrm{total}\_\mathrm{all}}}$	N_Δ_	$\frac{N_{\Delta }}{N_{\mathrm{total}\_\mathrm{all}}}$	Sen. (95% CI) MP	Sen. (95% CI) FloCo
I	18	17	1	5.6%	0	0	−1	−5.6%	100% (81.5–100%)	94.4% (72.7–99.9%)
II	77	68	0	0	9	11.7%	9	11.7%	88.3% (79.0–94.5%)	100% (95.3–100%)
III	82	71	5	6.1%	6	7.3%	1	1.2%	92.7% (84.8–97.3%)	93.9% (86.3–98.0%)
IV	274	240	7	2.6%	27	9.6%	20	7.3%	90.1% (86.0–93.4%)	97.4% (94.8–99.0%)
V	247	215	16	6.5%	16	6.5%	0	0	93.5% (89.7–96.3%)	93.5% (89.7–96.3%)
VI	110	96	8	7.2%	6	5.5%	−2	−1.8%	94.5% (88.5–98.0%)	92.7% (86.2–96.8%)
VII	175	161	3	1.7%	11	6.2%	8	4.6%	93.7% (89.0–96.8%)	98.3% (95.1–99.7%)
VIII	275	239	7	2.5%	29	10.6%	22	8.0%	89.5% (85.2–92.8%)	97.5% (94.8–99.0%)
Summary	1258	1107	47	3.7%	104	8.3%	57	4.5%	91.7% (90.1–93.2%)	96.3% (95.1–97.2%)

**Table 4:** The contrast-to-noise ratio (CNR) for each liver segment and lobe. The data are presented as the mean ± standard deviation. The respective *p*-values are stated. MP = monopolar, FloCo = flow-compensated.

Liver Segment	CNR (Mean ± Standard Deviation)						Liver Lobe
	MP	FloCo	*p*-Value	MP	FloCo	*p*-Value	
I	17.6 ± 10.2	22.8 ± 12.7	0.02	12.3 ± 8.9	15.9 ± 9.9	<0.001	left
II	9.4 ± 8.7	13.5 ± 8.8	<0.001				
III	11.8 ± 8.0	14.1 ± 8.9	<0.001				
IV	13.0 ± 8.9	16.7 ± 10.0	<0.001				
V	16.7 ± 9.9	17.4 ± 12.1	0.02	15.9 ± 10.1	18.1 ± 11.8	<0.001	right
VI	19.1 ± 12.6	21.7 ± 13.9	<0.001				
VII	13.3 ± 8.9	17.6 ± 11.2	<0.001				
VIII	15.6 ± 9.4	17.6 ± 10.9	<0.001				

## Data Availability

The original data presented in the study are openly available in GitHub at https://github.com/am-2309/FlowCompensatedDWI.git (uploaded and last accessed on 21 July 2025).

## Abbreviations

The following abbreviations are used in the manuscript:

CI	confidence interval
CNR	contrast-to-noise ratio
DWI	diffusion-weighted MRI
FLL	focal liver lesion
FloCo	flow-compensated
MP	monopolar
